# Identifying Anticancer Peptide via a Semi-Supervised Prediction Framework with Bio-Inspired Augmentation

**DOI:** 10.3390/biomimetics11100743

**Published:** 2026-10-15

**Authors:** Xuan Liu, Yuqing Yang, Tong Zhao

**Affiliations:** 1 School of Computer and Information Technology, Xinyang Normal University, Xinyang 464000, China; zhaotong@xynu.edu.cn; 2 School of Management, Xinyang Agriculture and Forestry University, Xinyang 464000, China; 2017220006@xyafu.edu.cn

**Keywords:** anticancer peptide prediction, drug discovery, data augmentation, deep learning, semi-supervised learning

## Abstract

Anticancer peptides (ACPs) have emerged as a promising class of therapeutic molecules due to their low toxicity and high specificity. However, experimentally identifying ACPs is labor-intensive and low-throughput, making virtual screening with intelligent models an attractive alternative. Despite recent progress, the number of ACP samples available in existing databases remains limited, which may cause predictive models to overfit the labeled information and exhibit suboptimal generalization. To address this issue, we propose a semi-supervised prediction framework with bio-inspired data augmentation for ACP identification, called AugACP. Specifically, we incorporate semantic knowledge into masked Transformer pretraining to guide ACP sequence augmentation and provide more reliable supervised signals. Based on the augmented data, we adopt a semi-supervised learning strategy that selects high-confidence pseudo-labels from augmented peptides to improve prediction performance. In the computational experiments, AugACP outperforms state-of-the-art baseline methods under various experimental configurations, particularly in generalizing to unseen oligopeptides. Moreover, t-SNE visualizations indicate that AugACP learns more discriminative embeddings for separating ACPs from non-ACPs, while external validation on novel ACPs also achieves an F1-score of 0.9272. These results highlight the predictive power of AugACP and its potential value in promoting anticancer drug discovery.

## 1. Introduction

Cancer is a complex disease that threatens human health, and identifying the right treatment for each patient is a critical challenge. With challenges in small-molecule drug development and increasing homogeneity in large-molecule drugs like antibodies, the search for novel and effective anticancer therapies has become urgent [1]. Anticancer peptides (ACPs) are short amino acid sequences with the potential to selectively target cancer cells while minimizing damage to normal healthy cells [2]. They are typically derived from natural proteins or synthetically designed using phage/yeast display technologies. However, even ACPs as short as 10 amino acids can give rise to as many as $10^{20}$ possible variants, in which therapeutic candidates are extremely rare. This enormous search space makes experimental ACP screening highly challenging and underscores the need for computational approaches to accelerate discovery.

In recent years, numerous computational methods have been proposed to classify peptides as ACPs or non-ACPs, primarily focusing on two aspects: peptide representation learning and activity prediction. In representation learning, early methods relied on hand-crafted features to construct the input space, such as amino acid composition [3], physicochemical properties [4], and k-mer frequency matrices [5]. Nowadays, natural language processing has gained increasing popularity, by treating amino acids as words and peptide sequences as sentences to extract latent embeddings [6]. For example, Lv et al. [7] built upon pretrained protein language models and integrated LSTM networks to derive sequence embeddings for ACPs. Guntuboina et al. [8] introduced a self-supervised BERT that leverages a masked residue reconstruction task to improve representation learning for ACP sequences. Li et al. [9] utilized Transformer encoders to capture contextual semantic embeddings from ACP samples. By contrast, structural representation learning for peptides is limited due to the scarcity of 3D conformations. A few studies have attempted to generate structures from sequences using tools like AlphaFold, and then transform them into residue or atomic graphs, so as to encode spatial and positional information via geometric deep learning [10]. In activity prediction, conventional methods have adopted machine learning algorithms (e.g., random forests, XGBoost, and ensemble learning), to determine whether a peptide exhibits anticancer activity [11,12]. Benefiting from the end-to-end capabilities of deep learning, recent advancements have devised various neural networks to optimize ACP representation and prediction [13]. For instance, Rao et al. [14] reformulated ACP prediction as a graph classification task and applied a graph convolutional network (GNN) to classify peptides based on residue co-occurrence patterns. Srivathsan et al. [15] adopted a contrastive learning strategy to align sequence and structure modalities into a shared space, then used a BERT decoder to predict ACP properties. Zhang et al. [16] deployed a convolutional neural network combined with the flow-attention mechanism to capture latent patterns from ACP sequences.

Despite these advances, ACP modeling is still hindered by the scarcity of labeled data. For instance, benchmark datasets such as CancerPPD [17] contain only thousands of known ACP sequences, which is far from sufficient for training robust models. Structural data are even harder to obtain due to the difficulty of peptide crystallization [18]. While some studies tried to incorporate AlphaFold-predicted 3D conformations to enhance structure representations, such strategies do not provide additional supervisory labels. Meanwhile, several works considered expanding the feature space by sampling non-ACPs from antimicrobial or antiviral peptides [19], but this cannot strengthen the positive signal specific to ACPs. In this context, data augmentation offers a promising way to enrich supervision by generating pseudo-samples, as has been widely explored in small- and macro-molecule fields [20]. Unlike image or text data, regular augmentations based on random perturbations, such as sequence mutation or structural noise addition, are unlikely to produce positive ACP instances, since even minor perturbations may cause substantial functional changes [21]. In fact, the amino acid arrangement and spatial configuration define ACP’s unique physicochemical properties. Typically, ACPs are enriched in positively charged lysine (K), arginine (R), histidine (H), and hydrophobic leucine (L), which impart cationic and hydrophobic characteristics and facilitate electrostatic interactions with anionic tumor cell membranes [22]. Moreover, the secondary structures of ACPs are predominantly α-helices and β-sheets, with β-turns and random coils occurring less frequently. Intrinsically disordered ACPs are often rich in glycine (G) and proline (P), while lacking well-defined secondary structures [23]. These insights reveal essential patterns within ACPs and provide a basis for biologically informed data augmentation to improve model generalization.

Along this line, we proposed a semi-supervised learning framework with bio-inspired data augmentation for predicting ACPs, called AugACP. Specifically, motivated by a priori knowledge from ACP libraries, we extract semantic information from peptide sequences to identify conserved regions. For the remaining non-conserved region, we deploy a masked Transformer for pretraining with AlphaFold-predicted structure constraints, guiding the generation of pseudo-labeled samples via amino acid mutations. After obtaining the augmented ACP data, we encode peptide sequences as ESM-2 [24] features and adopt a self-training semi-supervised learning [25] framework to improve ACP prediction. In this framework, a teacher decoder is first trained on the original labeled data and then used to assign pseudo-labels to the augmented data. High-confidence predictions of pseudo-labeled data are incorporated into the student decoder’s training process. This procedure is repeated iteratively, allowing the student model to refine its predictions progressively until convergence. To evaluate the proposed method, we conduct a comparison experiment on a large-scale benchmark ACP dataset. The results show that AugACP consistently outperforms existing state-of-the-art methods across multiple evaluation metrics, particularly in its ability to generalize to unseen oligopeptides. The t-SNE visualizations also indicate that AugACP produces more discriminative embeddings for separating ACPs from non-ACPs. In addition, we perform external validation on an independent database, and many predictions for unseen ACPs are further supported by published evidence. Together, these findings demonstrate AugACP’s robustness and highlight its potential for discovering novel anticancer therapeutic candidates.

## 2. Materials and Methods

### 2.1. Data Preparation

In this study, model training and testing for ACP prediction were conducted on five large-scale peptide databases: Peptihub [26], Dctpep [27], Apinapdb [28], Tumorhope [29], and CancerPPD 2.0 [30]. These resources contain experimentally validated ACPs and anticancer proteins from published articles, patents, and other public repositories. We performed data preprocessing to remove sequences containing non-canonical amino acids beyond the standard 20 types, as well as sequences longer than 80 amino acids, which are more likely to correspond to full-length proteins rather than peptides. Next, we merged ACP sequences from all five databases and removed duplicate sequences across the entire dataset. After that, the dataset comprised 5044 unique ACP sequences, as shown in Table 1. Since the source database provides only positive samples and lacks experimentally verified non-ACP (negative) instances, we adopted a negative sampling strategy following previous studies [15,31]. Given that true therapeutic peptides are rare relative to the vast sequence space, we randomly selected negative samples from the Human PeptideAtlas database [32], which contains over one million peptide sequences. Before negative sampling, sequences annotated as ACPs or overlapping with known ACP data were removed from Human PeptideAtlas. Therefore, the selected negative samples were considered non-ACP candidates based on the absence of reported anticancer activity annotations rather than experimentally validated inactive peptides. This resulted in a dataset with a positive-to-negative ratio of 1:5. We also examined sequence-level redundancy between ACPs and negative samples using Levenshtein distance. As shown in Supplementary Table S1, we observed sequence-level overlap between the two groups, indicating that the non-ACP candidates were not simply easy negatives. Meanwhile, only 1.17% of ACPs had highly similar counterparts (Levenshtein similarity ≥ 90%) in the negative samples, which suggests that the sampled negative set is unlikely to introduce substantial label noise. Further evaluation of potential dataset bias based on peptide length distribution and amino acid composition is provided in Supplementary Materials S1.

### 2.2. Model Architecture

#### 2.2.1. Problem Formulation

**Figure 1:**
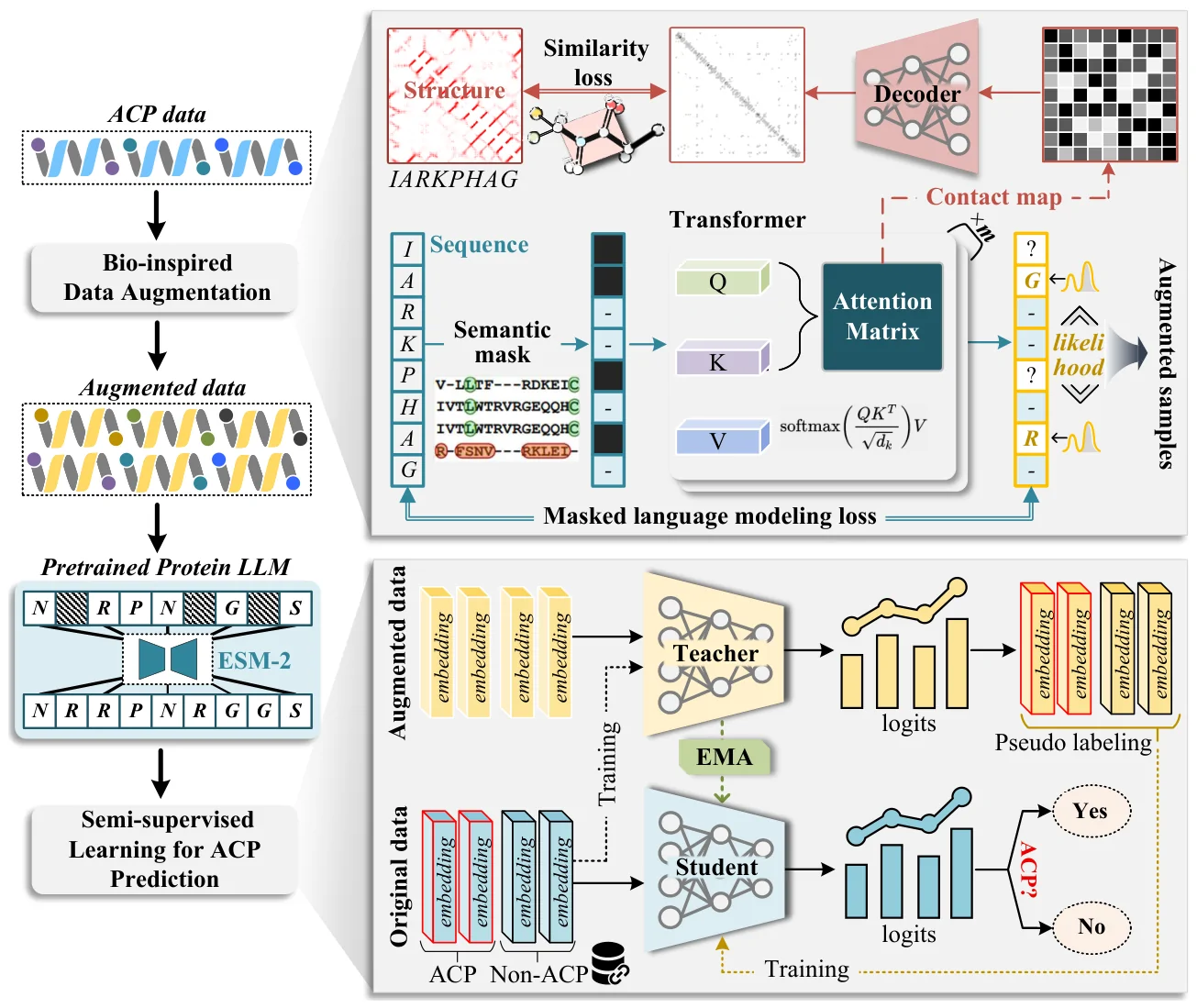
The architecture of AugACP consists of two main modules. (1) Bio-inspired Data Augmentation: Prior biological knowledge is used to identify conserved patterns in ACP sequences for semantic masking, which is further combined with a Transformer and contact map constraints to generate plausible augmented samples. (2) Semi-supervised learning for ACP prediction: Two decoders with identical architectures are instantiated as the teacher and student. The student is trained on the original labeled samples and the pseudo-labeled augmented samples generated by the teacher, while the teacher is updated through an exponential moving average (EMA) of the student’s parameters.

Let $D={\left(x_{i},y_{i}\right)}_{i=1}^{N}$ represent a labeled peptide dataset, where *N* is the sample size, $x_{i}$ is the input peptide, and $y_{i}\in \left\{0,1\right\}$ indicates whether the sample exhibits anticancer activity (with 1 for positive samples and 0 for negative samples). Our objective is to learn a mapping function that outputs a probability score p∈[0,1], where higher values indicate a greater likelihood of anticancer activity. To this end, we propose the AugACP model (illustrated in Figure 1), which consists of two main steps. (1) By leveraging biological knowledge from the ACP library, we implement a data augmentation strategy to generate additional samples, thereby expanding the limited data space. (2) Using the augmented data, we introduce a teacher–student semi-supervised learning framework. In this framework, ESM-2 encoded sequence features serve as inputs, and the teacher model generates pseudo-labels for the augmented samples, while the student model is trained on both augmented and real data to achieve robust ACP prediction. The following sections describe these two steps in detail.

#### 2.2.2. Bio-Inspired Data Augmentation

**Figure 2:**
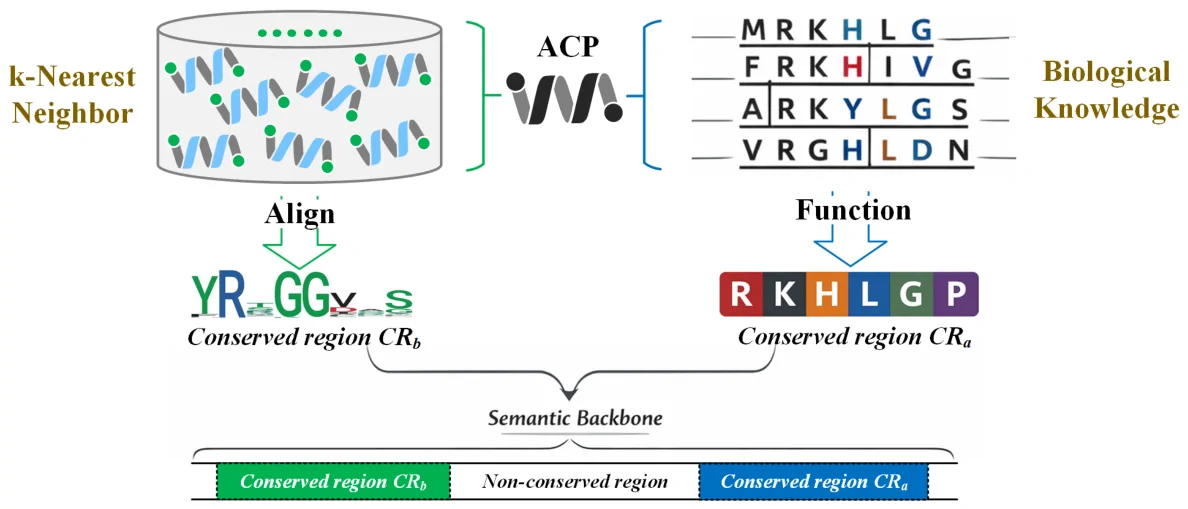
The process of selecting the conserved and non-conserved regions for ACP augmentation based on domain knowledge.

Generating pseudo samples that are closer to real ACPs is an intuitive way to strengthen the supervisory signal during model training. Given the biological complexity of peptides [33], we designed a reliable data augmentation strategy, ensuring that crucial amino acid (AA) sites remain unchanged during the mutation sampling process (Figure 2). Inspired by domain knowledge behind the ACP library [22], we focused on a biologically stable conserved region (${\mathrm{CR}}_{a}$) composed of arginine (R), lysine (K), histidine (H), leucine (L), proline (P), and glycine (G), owing to their high prevalence and important functional roles in existing ACPs. Specifically, R, K, and H contribute to electrostatic interactions with cancer cell membranes; L is associated with hydrophobic interactions, while G and P are frequently enriched in intrinsically disordered ACPs [22,23]. Here, “conserved” refers to residues that are expected to remain stable during augmentation due to their biological relevance, rather than strictly evolutionary conservation.

(1) $${\mathrm{CR}}_{a}\left(x_{i}\right)\leftarrow \left\{t|x_{i}\left[t\right]\in A\right\},\;A=\left\{R,K,H,L,P,G\right\}$$

For each ACP sequence $x_{i}$, we further identify its *k* nearest neighbors in the positive sample set ($D_{+}$) using the Levenshtein distance: $N\left(x_{i}\right)=\mathrm{TopK}{\left\{d_{\mathrm{Lev}}\left(x_{i},x_{j}\right)\right\}}_{j\neq i}$. We compare $x_{i}$ with each of its neighbors; if the amino acid at position *t* in $x_{i}$ aligns with that of more than a certain percentage δ of neighbors, it is considered a conserved site. These aligned sites are then grouped into a conserved region, denoted as ${\mathrm{CR}}_{b}$.

(2) $${\mathrm{CR}}_{b}\left(x_{i}\right)\leftarrow \left\{t|\frac{1}{k}\sum_{x_{j}\in N\left(x_{i}\right)}1\left(t\in \mathrm{LCS}\left(x_{i},x_{j}\right)\right)>\delta \right\}$$

where 1(·) is the indicator function, and LCS denotes the longest common subsequence. Together, two conserved regions (${\mathrm{CR}}_{a}$ and ${\mathrm{CR}}_{b}$) form the semantic backbone of ACP and represent keywords in the sequence sentence, while the remaining regions are viewed as “non-conserved” and assumed to have a lesser impact on ACP functionality. This design differs from random mutation or indel by distinguishing between ACP regions likely encoding functional invariants and those more open to variation.

Once the conserved region is identified, only positions outside this part are eligible for mutation. To do so, a masking operation is applied to the non-conserved regions, i.e., the corresponding amino acids are substituted with a mask token. Subsequently, masked sequences are represented using one-hot encoding, and an *m*-layer Transformer is trained to recover the masked positions and capture the semantic knowledge in their representations. Technically, for each amino acid token, three vectors are obtained after linear networks: q (query), k (key), and v (value). The attention score $\alpha_{ij}$ between any two amino acids (*i* and *j*) in the context is computed as follows:

(3) $$\alpha_{ij}=\mathrm{Softmax}(\left(q_{i}^{⊤}k_{j}\right)/\sqrt{\left|k\right|})$$

where the softmax function converts the similarity scores into probabilities. From this, an amino acid interaction matrix $A\in R^{n\times n}$ is obtained (*n* is the sequence length). The resulting embedding h is then fed into a multi-layer perceptron (MLP) to output the probability $\hat{p}\in R^{20}$ of all amino acids at the masked position:

(4) $$h_{i}=\sum_{j=1}^{n}\alpha_{ij}v_{j},\;\hat{p}\left(x_{i}|{\hat{x}}_{\setminus i}\right)=\mathrm{MLP}\left(h_{i}\right)$$

where ${\hat{x}}_{\setminus i}$ stands for the sequence excluding the masked position. The masked language modeling (MLM) [34] objective is trained using cross-entropy loss between the predicted amino acid type and the true one at the masked positions:

(5) $$L_{\mathrm{mlm}}=-E_{x\in D_{+}}\left[E_{\mathrm{mask}}\sum_{i\in \mathrm{mask}}\log p\left(x_{i}|{\hat{x}}_{\setminus i}\right)\right]$$

In addition to semantic knowledge, we expect the augmented samples to capture structural patterns inherent in ACP conformations. Hence, we regularize the attention matrix (A) with a contact map (M) derived from ACP structure. Structures are generated using the AlphaFold2 tool, and 3D coordinates of the backbone $C_{\alpha }$ atoms are utilized to construct the contact map, where each entry is assigned a value of 1 if the Euclidean distance between two $C_{\alpha }$ atoms is less than 8Å [35], and 0 otherwise. We minimize the similarity ($L_{\mathrm{sim}}$) between the contact map and the attention matrix to guide the prediction of amino acid probabilities $\hat{p}$. Concretely, we define the following augmentation loss:

(6) $$L_{\mathrm{aug}}=L_{\mathrm{mlm}}+\beta L_{\mathrm{sim}},\;L_{\mathrm{sim}}=-E_{x\in D_{+}}{\left[\sigma \left(A_{x}\right)-M_{x}\right]}^{2}$$

where σ is the sigmoid activation, E stands for the expectation, and β is a hyperparameter that balances the contribution of the regularization term. This term encourages the model not only to reconstruct masked sequence tokens, but also to generate substitutions consistent with the structural organization of peptides. After training, the MLP could output amino acid distributions at masked positions. Using the ranked likelihoods at these positions (i.e., the substitution probabilities for the 20 amino acids), we generate *l* pseudo-variants $\left({\tilde{x}}_{i}^{1},{\tilde{x}}_{i}^{2},\ldots ,{\tilde{x}}_{i}^{l}\right)$ per parent sequence. For each chosen site, the top-*l* amino acids with the highest likelihoods are used to replace the wild-type. As a result, we obtain a set of bio-inspired augmented samples $\tilde{D}$, which is used for the downstream ACP prediction training task.

#### 2.2.3. Semi-Supervised Learning for ACP Prediction

With the augmented peptide data, a naive approach is to treat all of them as positive labels (i.e., ACPs). However, this can easily bring in label noise and lead to overfitting, particularly when truly negative labels (i.e., non-ACPs) are misclassified as positive. Semi-supervised learning offers a more feasible solution by exploiting the additional information contained in unlabeled data. Based on this idea, we adopt a self-training semi-supervised learning paradigm, aiming to improve the model’s generalization by incorporating more reliable pseudo-label information.

First, to obtain informative sequence representations for downstream prediction, we employ ESM-2 [24], a Transformer-based protein language model pretrained on the UniRef [36] database. Benefiting from large-scale pretraining, ESM-2 captures rich evolutionary and contextual information from protein sequences. In detail, given an input sequence $\left[s_{1},s_{2},\ldots \right]$, we encode it using ESM-2 and apply average pooling over all amino acids to obtain a global embedding:

(7) $$z=\mathrm{AvgPooling}\left(\text{ESM-2}(\left[s_{1},s_{2},\ldots \right])\right)$$

Based on the extracted embedding, we construct a teacher–student framework, where both of them are implemented as MLP decoders with the same architecture. The teacher decoder ($f_{T}$) is first trained on the labeled dataset $D={\left\{\left(x_{i},y_{i}\right)\right\}}_{i=1}^{N}$ under the binary cross-entropy loss:

(8) $$L_{T}=-\frac{1}{N}\sum_{i=1}^{N}\left[y_{i}\log g_{i}^{T}+\left(1-y_{i}\right)\log\left(1-g_{i}^{T}\right)\right]$$

where $g^{T}=f_{T}\left(z\right)$ denotes the predicted logit and *y* is the corresponding ground-truth label. Subsequently, the trained teacher decoder is used to generate pseudo-labels for the augmented unlabeled samples $\tilde{D}={\left\{{\tilde{x}}_{j}\right\}}_{j=1}^{N^{\prime }}$. To mitigate noise and prevent error propagation, only high-confidence predictions are retained:

(9) $${\tilde{y}}_{j}=\left\{\begin{array}{cc} 1, & g_{j}^{T}\geq \tau_{+},\\ 0, & g_{j}^{T}\leq \tau_{-},\\ \mathrm{discarded}, & \tau_{-}<g_{j}^{T}<\tau_{+}, \end{array}\right.$$

where $\tau_{+}$ and $\tau_{-}$ are the positive and negative confidence thresholds, respectively. The selected pseudo-labeled samples are merged with the original labeled set to form an expanded training set: $D^{*}=D\cup \left\{\left({\tilde{x}}_{j},{\tilde{y}}_{j}\right)\right\}$. The student decoder $f_{S}$ is then trained on $D^{*}$, allowing it to learn from both real and pseudo-labeled samples.

Finally, a mean teacher paradigm [37] is utilized to stabilize the above teacher–student iterative process. Instead of updating the teacher model via backpropagation at each step, its parameters are updated as the exponential moving average (EMA) of the student parameters:

(10) $$\Theta_{t}^{T}=\omega \Theta_{t-1}^{T}+\left(1-\omega \right)\Theta_{t}^{S}$$

where ω∈[0,1] is the EMA coefficient, $\Theta_{t}^{S}$ is the student parameters at step *t*, $\Theta_{t-1}^{T}$ is the teacher parameters at step t−1. In this manner, the teacher provides more stable supervision for pseudo-label generation, while the student progressively improves through training on the expanded dataset. Finally, the trained student decoder is used for ACP prediction on the testing set.

## 3. Results

### 3.1. Evaluation Protocol

To evaluate model performance, the dataset was first randomly divided into a training set (80%) and a testing set (20%), with no overlap between them. A 5-fold cross-validation (5-CV) scheme was then applied to the training set, which was split into five equally sized subsets. In each iteration, four subsets were used for model training and the remaining one for validation, ensuring each subset served as the validation set once. The model achieving the best validation performance in each fold was subsequently assessed on the testing set. For our binary ACP prediction, performance was measured using four standard metrics: area under the curve (AUC), area under the precision–recall curve (AUPR), accuracy (ACC), and F1-score (F1). Note that sequence augmentation, nearest-neighbor selection, AlphaFold2 structure generation, and model optimization were performed exclusively using the training set. The testing set was kept completely separate during training and used only for final performance evaluation.

In this study, we benchmark AugACP against six established ACP prediction baselines (including both sequence-based and structure-based methods), as described below:

- **AACFlow** [16] combined multi-layer convolutional neural networks to capture both global and local sequence information. A multi-head flow-attention mechanism is introduced to extract deep features for improving ACP predictions.
- **ACP-MS** [12] converted peptide sequences into a digital feature set, using the AdaBoost model to select the most discriminative features. A stochastic gradient descent classifier is then applied to identify ACPs and non-ACPs.
- **ACPred** [11] developed an interpretable model based on handcrafted sequence features and classical machine learning algorithms, using SVM for ACP classification and random forest for feature importance analysis.
- **ACP-EPC** [38] employed a dual-stream framework that integrates ESM-2 embeddings with handcrafted physicochemical features, and utilized a cross-attention layer to align contextual residue information with conventional descriptors for ACP prediction.
- **Multi-Peptide** [15] used the transformer-based language model and a graph neural network encoder to learn complex representations of ACP sequences, followed by a CLIP-based alignment between sequence and structural features.
- **ACPScanner** [13] incorporated sequential, physicochemical, and secondary structural information to build a feature space. It first used a LightGBM classifier to predict ACPs and non-ACPs, and then inferred various targeted cancer types.

### 3.2. Hyperparameter Settings

We determined model hyperparameters through extensive trials on the training set using 5-CV, with no information from the testing set involved. In the bio-inspired data augmentation, the number *k* of nearest neighbors used to calculate the edit distance is set to 5. The transformer encoder used for mask pretraining is configured with m=3 layers and 4 attention heads. The percentage δ in conserved region ${\mathrm{CR}}_{b}$ is defined as 0.5. Mutation sites in the non-conserved regions are selected randomly, where the number of mutation sites ranges from 2 to 3. We set the coefficient (β), which balances the structural constraint task, to 1. Our augmentation ratio *l* is 1:5, meaning that each ACP sequence is expanded into five pseudo-samples. The configuration of AlphaFold2 used for peptide structure generation is provided in the Supplementary Materials S2. In the semi-supervised learning for ACP prediction, we adopt a lightweight version of ESM-2 (i.e., ESM2_t33_650M) to generate sequence representations, where each amino acid token is embedded as a 1280-dimensional vector. Both the teacher and student decoders consist of a 3-layer MLP with dropout (0.2) and batch normalization. The positive and negative thresholds ($\tau_{+}$) and ($\tau_{-}$) for pseudo-labeling the augmented samples are set to 0.9 and 0.1, respectively. The EMA coefficient is fixed at 0.99. Finally, the entire model is optimized using the Adam optimizer with a learning rate of 0.0001 and a batch size of 128. Detailed hyperparameter settings can be found in the source code (https://github.com/liuxuan666/AugACP), accessed on 10 October 2026. For the baseline methods, we followed the original implementations and applied them to our datasets and ACP prediction tasks, using either the best or default hyperparameters.

### 3.3. Method Performance Comparison

**Figure 3:**
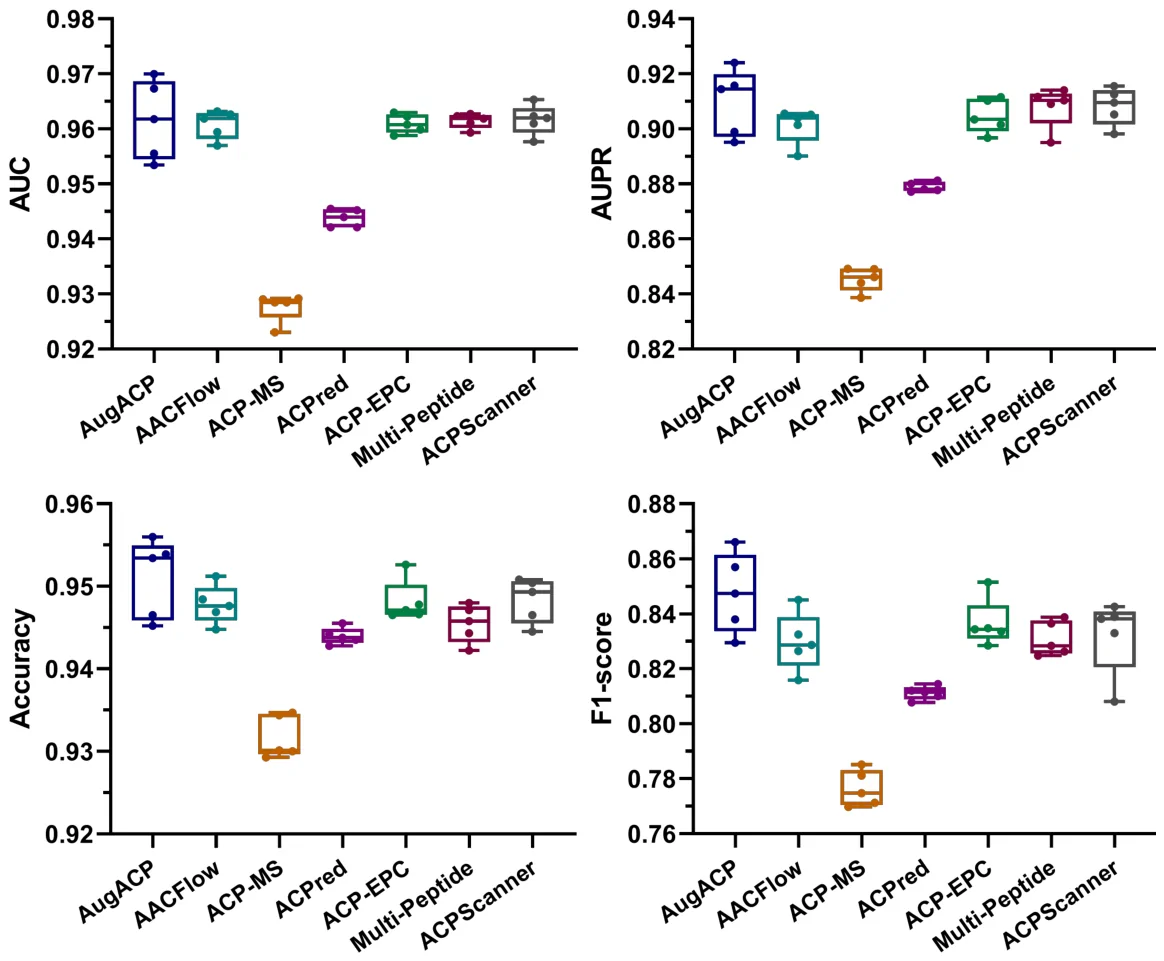
Performance comparison of all methods in terms of AUC, AUPR, Accuracy, and F1-score, where the boxplots show the performance distributions across the 5-CV runs on the testing set.

Figure 3 illustrates the performance results of all methods across four metrics on the testing set. In terms of AUC, AUPR, and F1-score, our AugACP outperforms all baseline methods, achieving mean values of 0.9616, 0.9084, and 0.8475, respectively. While AugACP demonstrates a modest lead in AUC, its superior performance in F1-score and AUPR is particularly notable, given the imbalanced nature of our dataset (the number of negative samples is five times that of positive samples). We also observed relatively high variance in our method during 5-CV, which may be attributed to bias introduced by augmented data and pseudo-labels used in mean teacher learning. Furthermore, the performance of the two methods, ACP-MS and ACPred, was significantly lower than that of the other baselines. Both of them rely on traditional machine learning algorithms, which may be less effective than deep learning models in capturing the complex representations of peptides. Additionally, baseline methods that rely solely on sequence data and those that incorporate structural information showed minimal performance differences. This is likely due to the fact that peptide structures were predicted by AlphaFold-2 rather than obtained through experimental measurements, and the ACP samples typically contain fewer amino acids, impeding the contributions of structural information.

Although identical sequences were removed during dataset preprocessing, highly similar ACPs may still exist between the training and testing sets due to the limited diversity of short peptides. To evaluate model robustness under a stricter sequence similarity constraint, we constructed a homology-reduced subset by removing testing ACPs with high similarity (≥80% sequence identity) to the training set based on CD-HIT clustering. The results of AugACP and baseline methods on this subset are summarized in Supplementary Table S2. Although all methods showed a slight decrease in this setting, our AugACP consistently outperformed the baselines, supporting its robustness under reduced sequence homology.

### 3.4. Ablation Results

To evaluate the necessity of each component in our model framework, we conducted a series of comparisons between AugACP and its variants. The ablation results on the testing set are presented in Table 2:

- AugACP without data augmentation (w/o DA): We remove the data augmentation process and train the predictor using only the original labeled dataset.
- AugACP without conserved region *a* (w/o ${\mathrm{CR}}_{a}$): We remove the conserved region that consists of six residues (R, K, H, L, G, P) during data augmentation.
- AugACP without conserved region *b* (w/o ${\mathrm{CR}}_{b}$): We remove the conserved region identified by nearest neighbors and LCS alignment during data augmentation.
- AugACP without semantic knowledge (w/o SK): We replace semantic augmentation with random site mutations, without distinguishing between conserved and non-conserved regions.
- AugACP without contact map (w/o CM): We remove the regularization loss $L_{\mathrm{sim}}$, which is constructed using the contact map during masked language pretraining for data augmentation.
- AugACP without ESM-2 features (w/o ESM): We omit the use of ESM-2 for representation extraction, replacing it with another protein language model: ProtTrans [39].
- AugACP without mean teacher updating (w/o MTU): We exclude the exponential moving average (EMA) update, where the student and teacher decoders no longer share parameters.

**Table 2:** Average results of ablation experiments.

Variants	AUC	AUPR	Accuracy	F1-Score
AugACP	0.9616	0.9084	0.9510	0.8475
(w/o DA)	0.9575	0.9024	0.9442	0.8252
(w/o ${\mathrm{CR}}_{a}$)	0.9610	0.9058	0.9445	0.8330
(w/o ${\mathrm{CR}}_{b}$)	0.9605	0.9096	0.9465	0.8380
(w/o SK)	0.9628	0.9003	0.9314	0.8178
(w/o CM)	0.9607	0.9036	0.9460	0.8446
(w/o ESM)	0.9588	0.9023	0.9370	0.8256
(w/o MTU)	0.9629	0.9079	0.9465	0.8355

As observed, removing the data augmentation step (w/o DA) significantly degraded the performance of AugACP across all metrics. This highlights the importance of additional training signals, even when using pseudo-labeled samples. Next, the results from the variants AugACP (w/o ${\mathrm{CR}}_{a}$) and (w/o ${\mathrm{CR}}_{b}$) showed that both types of semantic-aware site selection are crucial for helping the model select better augmented samples. The results of AugACP (w/o SK) show that semantic knowledge-guided augmentation is more effective than random mutation. When this component was removed, the Accuracy and F1-score decreased from 0.9510 and 0.8475 to 0.9314 and 0.8178, respectively. Furthermore, the lower performance of AugACP (w/o ESM) highlights the advantage of ESM-2 representations over ProtTrans, which may be attributed to ESM’s larger-scale pretraining and stronger ability to capture contextual sequence information. The mean teacher paradigm block also played a vital role in our framework. This is evidenced by the performance of AugACP (w/o MTU), which showed a noticeable decline when EMA updating was removed, preventing the student and teacher decoders from sharing parameters. In conclusion, every module is essential for effective ACP prediction, and removing any of them results in reduced performance.

### 3.5. Sensitivity and Visual Analysis

**Figure 4:**
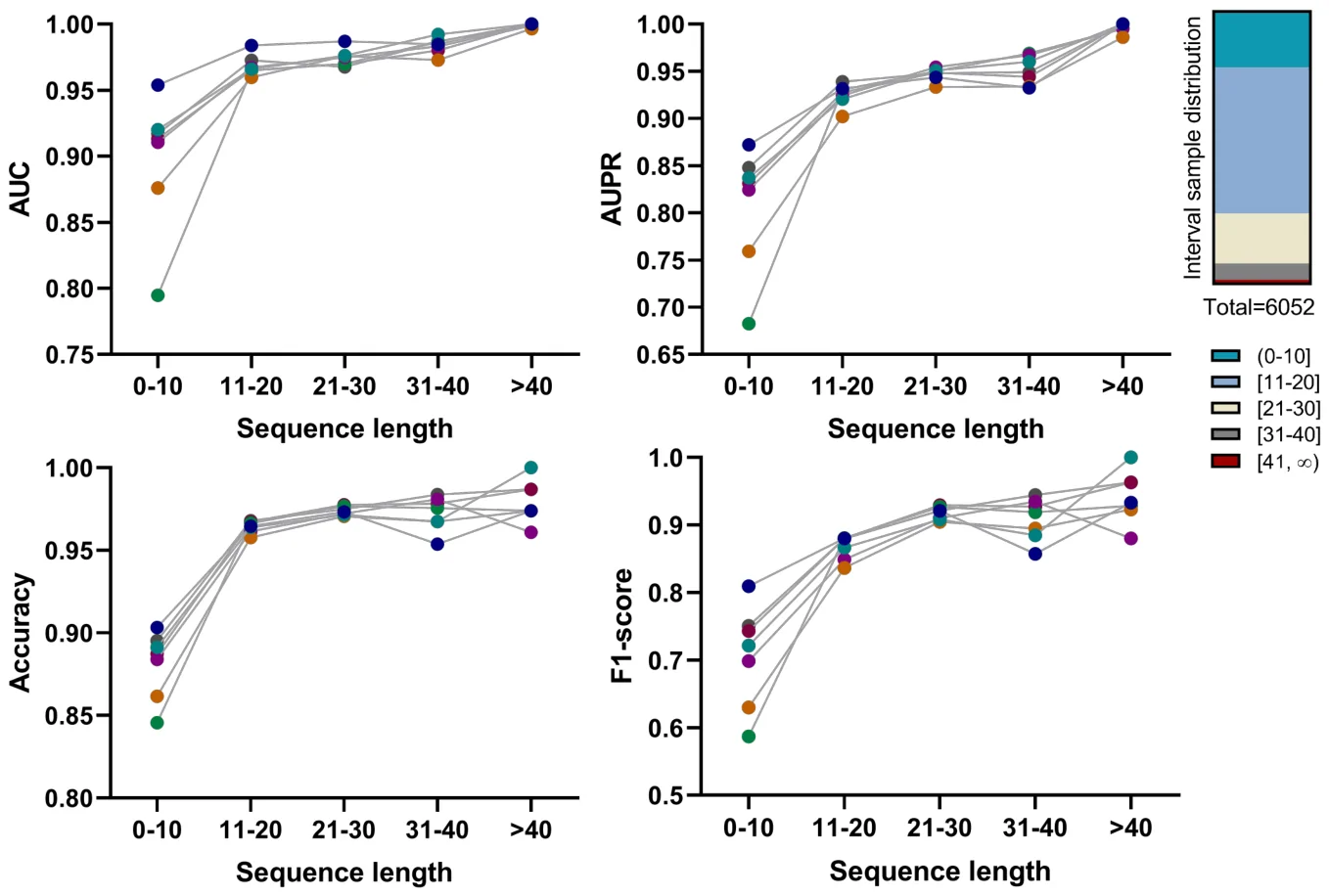
Average prediction performance of the models across different ACP sequence length intervals (i.e., 0–10, 11–20, 21–30, 31–40, and >40) and the corresponding sample distribution (i.e., legend on the right).

In our dataset, ACP sequence lengths range from 2 to 80 amino acids, resulting in a highly heterogeneous distribution that may complicate model learning. To investigate model specificity, we categorized the ACP samples into five intervals based on sequence length, as shown in Figure 4. The results indicated that AUC, AUPR, accuracy, and F1-score all increase with sequence length. One possible explanation for this trend is that longer peptides exhibit clearer patterns that are easier for the model to identify, while shorter peptides, with less sequence information, are harder to model. Notably, the figure also showed that the prediction accuracy for oligopeptides in the 1–10 amino acid range is significantly lower, and the performance across all methods for peptides in the 11–40 amino acid range is relatively consistent. In particular, for the method AACFlow, the CNN it uses seemed less capable of capturing the amino acid correlations in short peptides, which led to the lowest performance. We also analyzed the sample size within each length interval and found no clear linear relationship between the number of samples and prediction performance. These results suggest that sequence length, rather than sample count alone, is an important factor affecting ACP prediction.

**Figure 5:**
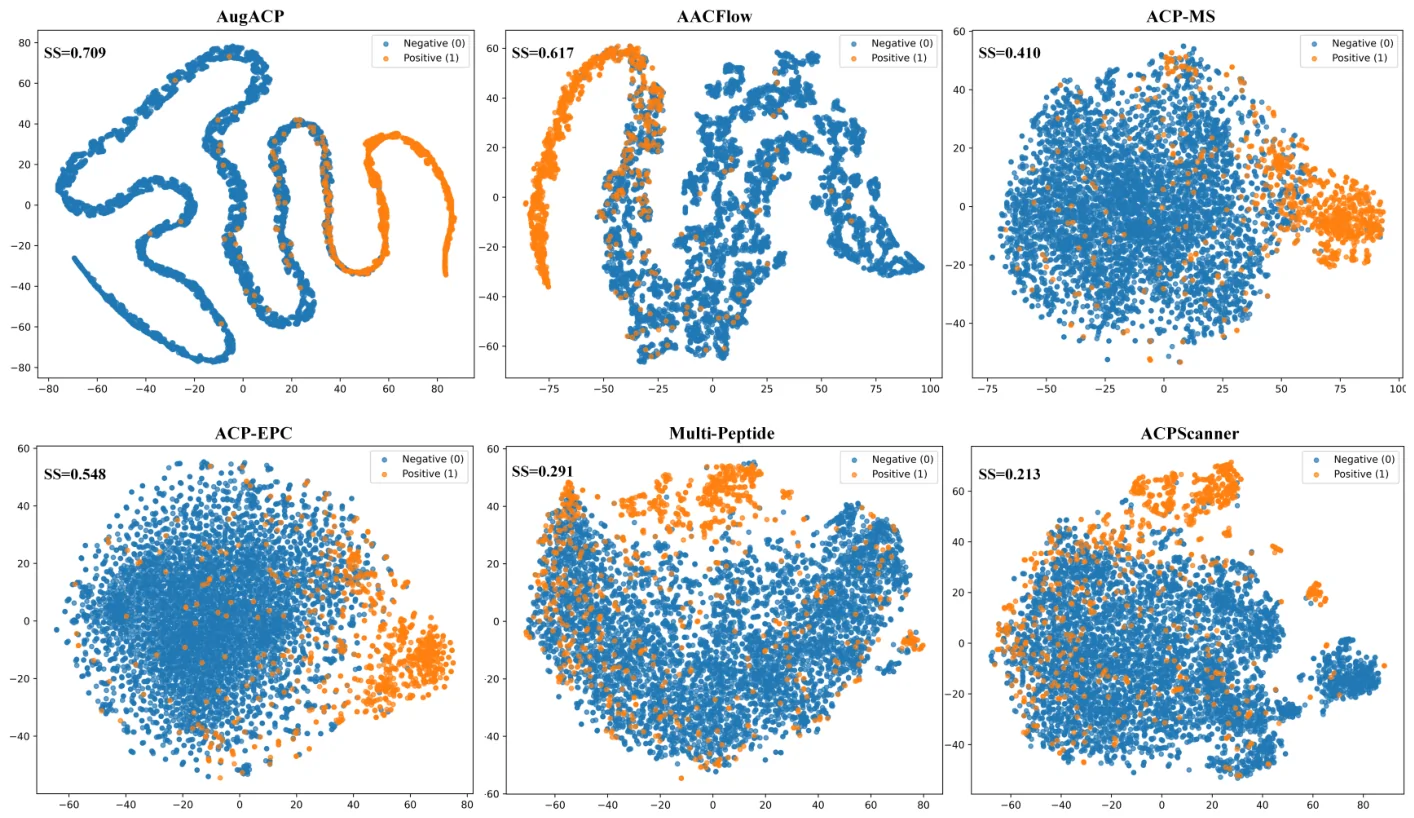
The t-SNE visualization of embeddings learned from AugACP and 5 baselines (AACFlow, ACP-MS, AAC-EPC, Multi-Peptide, and ACPScanner), where all embeddings are extracted from the last layer of each model.

Since the effectiveness of deep learning models largely depends on the quality of representation learning, we further investigated its impact on prediction performance. Specifically, we visualized the peptide embeddings learned during training, excluding ACPred because it uses a traditional SVM rather than deep learning. Using the testing set as an example, the embeddings were projected into a 2D space via t-SNE [40], as shown in Figure 5. The results show that several methods are able to separate the majority of non-ACPs from the minority of ACPs in the embedding space. Notably, AugACP and AACFlow exhibit a clearer and more structured distribution, suggesting their ability to capture more complex patterns in peptide data. To quantitatively evaluate this observation, we further computed silhouette scores (SS) [41] based on the learned embeddings (before t-SNE projection), which measure intra-cluster compactness and inter-cluster separation. In Figure 5, AugACP achieves superior discriminative capability (SS = 0.709) compared to the baselines. In addition, we visualized the initial representations encoded by ESM-2 using t-SNE, and calculated its silhouette score (Supplementary Figure S1). AugACP and several baseline methods (e.g., AACFlow and ACP-MS) showed a better ability to distinguish ACPs from non-ACPs than the initial ESM-2 representations (SS = 0.252). Overall, these findings confirm AugACP’s effectiveness in learning informative representations for ACP identification.

### 3.6. External Verification for Screening Novel ACPs

**Figure 6:**
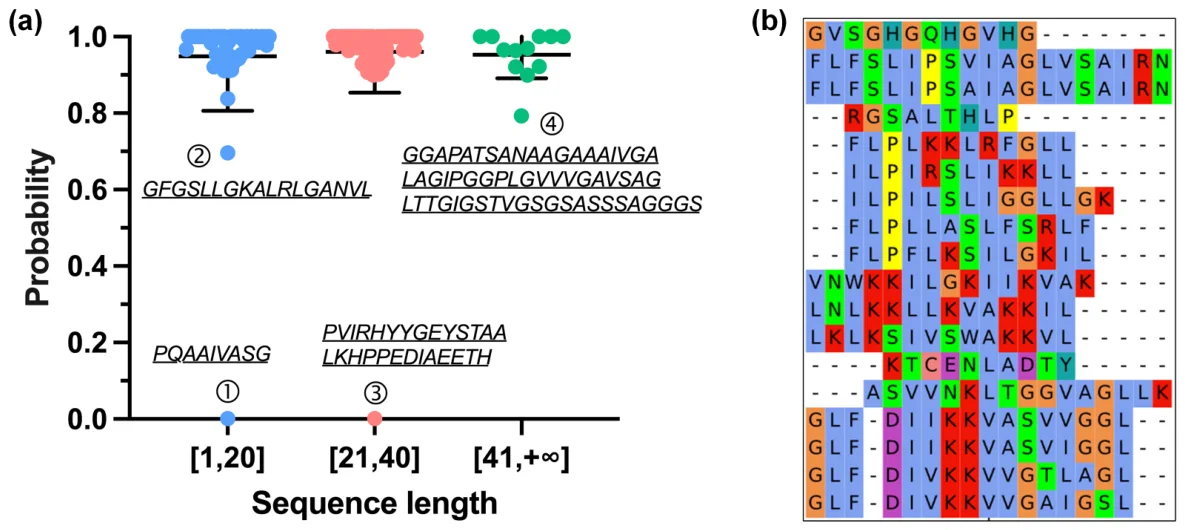
Statistical analysis of external validation. (**a**) Distribution of model-predicted ACP probabilities across sequence-length intervals, where each dot represents the predicted probability that an external sample is an ACP. The four marked points indicate representative low-probability samples. (**b**) Multiple sequence alignment of external validation samples identified as ACPs by our model (probability > 0.5) with sequence lengths in the interval of [10, 20].

To examine whether AugACP can identify novel ACPs beyond the original data distribution, we conducted external verification using two independent databases, SATPdb [42] and APD6 [43], which store hundreds of therapeutic peptides with antitumor activity that are absent from our dataset. Here, external sequences identical or highly homologous to those in the original ACP dataset were removed using CD-HIT with an 80% identity threshold. From the remaining resources, we collected 156 external ACP samples across different sequence-length intervals, including 54 samples of length 1–20, 91 samples of length 21–40, and 11 samples longer than 40 amino acids. The prediction results across three intervals are shown in Figure 6a. Since this external set contains only positive samples, higher predicted probabilities indicate better model confidence. Overall, AugACP performed well on the external data, though a few samples received relatively low-confidence predictions. We further visualized the samples with sequence lengths between 10 and 20, and found no obvious common patterns in the sequence alignment results (Figure 6b). This suggests that AugACP generalizes well and can recognize ACPs with diverse amino acid combinations. Nevertheless, our AugACP failed on two ACP samples shown in Figure 6a: sequence 1 (“PQAAIVASG”, named emericellipsin A) and sequence 3 (“PVIRHYYGEYSTAALKHPPEDIAEETH”, named Boma-CATH). Previous in vitro studies showed that emericellipsin A exhibits selective cytotoxicity against HepG2 and HeLa cell lines [44], while Boma-CATH inhibits A375 cell proliferation and migration through dual antitumor mechanisms (pyroptosis induction and metastasis suppression) [45]. These cases show that some ACPs remain challenging to identify, especially when their sequence patterns differ from those commonly observed in the training data.

Moreover, we constructed a more comprehensive external validation set by incorporating independent negative samples collected from Human PeptideAtlas, so as to evaluate the practical classification capability of AugACP. This negative set contained twice as many samples as the external ACP set, with no reported ACP annotations and no sequence overlap with the original dataset. We also assessed potential dataset bias based on peptide length distribution and amino acid composition, as detailed in Supplementary Materials S1. As shown in Supplementary Table S3, AugACP achieved an F1-score of 0.9272 and maintained superior discrimination performance compared with two representative baseline methods (Multi-Peptide and ACPScanner), which further supports its robustness on independent external data.

## 4. Conclusions

In recent years, AI-driven ACP screening has advanced substantially through the integration of techniques such as Transformers, pretraining, and multimodal learning. To address the current shortage of labeled ACP data, we propose AugACP, a deep learning framework that first performs semantic knowledge-guided data augmentation and then employs semi-supervised learning for ACP prediction. The key idea is to generate high-quality pseudo-samples that better approximate the true ACP distribution, improving model generalization. We evaluated AugACP on a comprehensive benchmark dataset, and the experimental results show that it outperforms existing state-of-the-art methods, particularly on oligopeptide samples. Despite these gains, AugACP still has certain limitations, especially in modeling structural information. Efficiently encoding structural features with geometric deep learning [46] in the presence of a large number of negative samples remains challenging. Yet we believe AugACP has great potential to accelerate the discovery of novel ACP-based anticancer therapeutics.

## Figures and Tables

**Table 1:** Positive Sample Number of Different Sources in Our Dataset.

Database	Sample Number	Proportion
Peptihub [26]	148	(2.93%)
Dctpep [27]	3410	(67.61%)
Apinapdb [28]	93	(1.84%)
CancerPPD 2.0 [30]	1125	(22.30%)
Tumorhope [29]	268	(5.31%)

## Data Availability

The databases used in this study are all publicly available. All source data and code related to this work are freely available at https://github.com/liuxuan666/AugACP, accessed on 10 October 2026.

## Supplementary Materials

The following supporting information can be downloaded at https://www.mdpi.com/article/10.3390/biomimetics11100743/s1. Table S1: Sequence redundancy percentage between ACPs and negative samples using Levenshtein distance; Table S2: Performance comparison on the homology-reduced subset; Table S3: Performance comparison on an external validation containing negative samples; Table S4: Peptide length distribution analysis; Table S5: Proportion distribution of amino acid composition; Figure S1: The t-SNE visualization in the initial representation space, where SS is the silhouette score, and Positive/Negative denotes ACP/non-ACP; S1: Negative-set and source bias; S2: AlphaFold2 configuration.
