# Supplementary material for: Identifying Anticancer Peptide via a Semi-Supervised Prediction Framework with Bio-Inspired Augmentation
Source: Biomimetics (Basel). 2026 Oct 15;11(10):743. doi: 10.3390/biomimetics11100743 (PMC13702777; doi:10.3390/biomimetics11100743)
Supplement: Supplementary file 1 [file biomimetics-11-00743-s001.zip › biomimetics-4570161-supplementary.pdf]

Supplementary Materials

Table S1. Sequence redundancy percentage between ACPs and negative samples using Levenshtein distance

Comparison	Levenshtein similarity	Percentage
Pos vs. Neg (ACP vs. Negative sample)	= 100%	0.0%
	≥ 90%	1.17%
	≥ 80%	3.62%
	≥ 70%	10.61%
	≥ 60%	36.16%
	≥ 50%	84.85%

Note: Levenshtein similarity is defined as $(1 - D/L) \times 100\%$*, where* D *represents the editing distance between two peptide sequences and* L *represents the maximum sequence length.*

Table S2. Performance comparison on the homology-reduced subset

Method	AUC	AUPR	Accuracy	F1-score
AugACP	0.9524	0.8949	0.9466	0.8280
AACFlow	0.9474	0.8773	0.9336	0.8070
ACP-MS	0.9086	0.8369	0.9305	0.7492
ACPred	0.9336	0.8520	0.9352	0.7884
ACP-EPC	0.9501	0.8680	0.9327	0.8029
Multi-Peptide	0.9457	0.8871	0.9414	0.8115
ACPScanner	0.9418	0.8802	0.9255	0.8048

Table S3. Performance comparison on an external validation containing negative samples

Method	AUC	AUPR	Accuracy	F1-score
AugACP	0.9637	0.9550	0.9546	0.9272
Multi-Peptide	0.9523	0.9486	0.9419	0.9052
ACPScanner	0.9490	0.9378	0.9347	0.9011

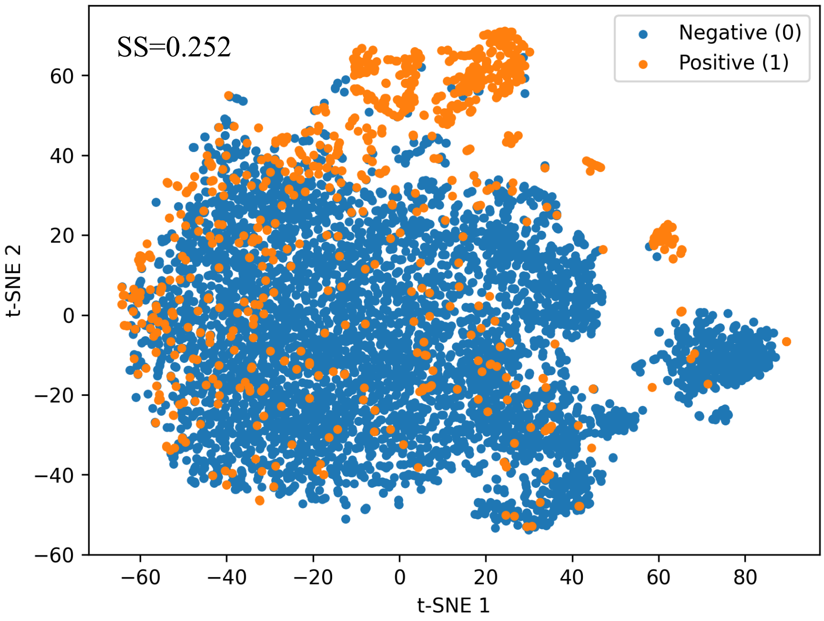

Figure S1. The t-SNE visualization in the initial representation space, where SS is the silhouette score, and Positive/Negative denotes ACP/non-ACP.

S1: Negative-set and source bias

To further evaluate potential database-source bias between ACP and non-ACP samples, we analyzed basic sequence characteristics, including peptide length distribution and amino acid composition. As shown in Table S4, ACPs and non-ACPs exhibited similar peptide length distributions in original data, suggesting that sequence length differences were unlikely to be the primary factor influencing model performance. Amino acid composition analysis (Table S5) revealed largely consistent overall patterns between ACPs and non-ACPs, with several variations in individual amino acid frequencies. These observations indicate that the predictive performance of our model is not solely attributed to simple sequence descriptors.

Furthermore, we evaluated the additional negative validation set using the same sequence characteristics. Compared with the original non-ACP data, the additional negative set showed similar but not identical peptide length distributions and variations in amino acid composition profiles, reflecting inherent sequence diversity while maintaining a comparable negative peptide distribution. This analysis supports the reliability of the additional negative validation set for evaluating model generalization.

Table S4. Peptide length distribution analysis

Data	Mean length	Median length	Std
ACP (Original data)	16.99	15	9.41
non-ACP (Original data)	17.04	15	8.73
non-ACP (Additional set)	16.11	16	8.22

Table S5. Proportion distribution of amino acid composition

Amino acid type	Original ACPs (%)	Original non-ACPs (%)	Additional non-ACPs (%)
A	7.47	7.64	7.73
C	3.96	2.53	1.59
D	2.52	4.94	5.69
E	2.7	3.07	6.06
F	4.92	3.31	3.58
G	6.89	7.34	7.2
H	1.91	2.52	2.05
I	5.93	4.33	3.25
K	12.93	10.28	9.88
L	11.25	9.7	8.48
M	1.28	1.72	1.36
N	2.55	3.86	3.9
P	4.39	6.83	6.14
Q	2.39	4.06	4.84
R	10.18	7.28	3.84
S	4.77	5.05	8.9
T	3.46	4.58	5.08
V	4.77	6.28	6.73
W	3.16	1.92	0.87
Y	2.57	2.76	2.83

● S2: AlphaFold2 configuration

In this study, peptide structures were predicted using ColabFold (v1.5.5), an accelerated implementation of AlphaFold2 deployed on a local Ubuntu system. Peptide sequences were formatted into FASTA files and used as input for structure prediction. The prediction was performed using the following parameters:

- **Number of generated models (--num-models): 5**
  Five independent structural models were generated for each peptide sequence to sample possible conformational states.
- **Number of recycling iterations (--num-recycle): 3**
  Three recycling iterations were applied during structure prediction to iteratively refine the predicted structures.
- **Early stopping criterion (--stop-at-score): 75**
  The prediction process was terminated when the confidence score reached the

specified threshold.

- **Template search (--templates) and Amber relaxation (--amber) were not enabled.**

After prediction, the generated structures were evaluated based on predicted Local Distance Difference Test (pLDDT) scores, which reflect residue-level local confidence of AlphaFold2 predictions. The highest-ranked model according to ColabFold confidence-based ranking was selected as the representative structure for downstream tasks.
